# Thyroid reoperation using intraoperative neuromonitoring

**DOI:** 10.1007/s12020-017-1443-x

**Published:** 2017-10-19

**Authors:** Beata Wojtczak, Krzysztof Sutkowski, Krzysztof Kaliszewski, Marcin Barczyński, Marek Bolanowski

**Affiliations:** 1 Department and Clinic of General, Gastroenterological and Endocrine Surgery, Wroclaw Medical University, Wroclaw, Poland; 2 Department of Endocrine Surgery, Third Chair of General Surgery, Jagiellonian University Medical College, Kraków, Poland; 3 Department of Endocrinology, Diabetes and Isotope Therapy, Wroclaw Medical University, Wroclaw, Poland

**Keywords:** Thyroid reoperation, Intraoperative neuromonitoring, Recurrent laryngeal nerve

## Abstract

**Purpose:**

Thyroid reoperations are at a high risk of recurrent laryngeal nerve (RLN) injury. The aim of the study was to investigate whether the use of intraoperative neuromonitoring **(**IONM) can aid in the RLN identification and minimize the risk of its injury, in comparison with visual RLN identification.

**Methods:**

This was a retrospective cohort study of patients who underwent thyroid reoperations with and without the use of IONM. Primary endpoint was the RLN identification rate; secondary: the prevalence of RLN injury, the frequency of total thyroidectomies, and the course of the RLN.

**Results:**

The study involved 61 patients undergoing thyroid reoperation among whom 24 were operated on with visual RLN identification only, while 37 procedures used IONM. In the non-monitored reoperations, 44.4% of the RLN were visually identified, as opposed to 91.6% in the IONM group (*p* < 0.001). Transient paresis occurred in three nerves with visualization (6.6%), and in one in IONM group 1.6% (*p* = 0.185). Permanent paresis occurred in the group with visualization (6.6%), as opposed to none with neuromonitoring. The extent of resection in both groups was significantly different (*p* = 0.043). Total, near-total thyroidectomies, Dunhill operations and subtotal thyroidectomies were performed in 71, 17, 4, and 8% in the visualization group, and in 94, 0, 3, and 3%, respectively, in the IONM group. A non-anatomical RLN course was observed in 80% of the reoperations with IONM.

**Conclusions:**

Thyroid reoperation should be performed using IONM, because it allows for a significantly improved RLN identification rate and a significantly more radical resection.

## Introduction

The indications to thyroid surgery and the extent of the initial thyroid resection influence postoperative outcomes. Nowadays, more and more, total thyroidectomy seems to be the best surgical treatment option in most thyroid pathologies [1–4]. However, it is still the case that not all thyroid operations are sufficiently radical, and reoperations are frequently required. There are two main indications for reoperative thyroid surgery: recurrence of a multinodular goiter (or Graves’ disease) many years after the first operation, or when cancer is recognized postoperatively after a non-total thyroidectomy [5]. Thyroid reoperations are always at an increased risk of complications, in particular recurrent laryngeal nerve (RLN) injury and hypoparathyroidism [5, 6]. The prevalence of RLN palsy ranges from 2 to 30% [6–9], and is much higher than in initial thyroid surgery [10]. Thomusch et al. calculated a relative risk of 3.1 for RLN palsy in thyroid reoperations compared with primary benign goiter surgery [8]. Reoperative thyroid surgery is difficult for young and inexperienced surgeons, but even skilled and experienced surgeons can have difficulty mapping the RLN and preventing inadvertent RLN injury, particularly in non-anatomical fields with many scars from the first operations. Many techniques and strategies are recommended in reoperations to prevent RLN palsy [5, 11], and one of them is the use of intraoperative neuromonitoring (IONM). IONM is already a standardized technique [12, 13], and is accepted by many endocrine and head and neck surgeons, particularly in difficult thyroid operations [14]. The aim of this study was to investigate whether the use of IONM can aid in the RLN identification and minimize the risk of nerve injury in thyroid reoperations.

## Material and methods

This was a retrospective cohort study of patients who underwent thyroid reoperations with and without the use of IONM. The database of thyroid surgery was searched for eligible patients treated in 2011–2014. The outcomes of reoperative procedures with and without the use of IONM were compared. Primary endpoint was the RLN identification rate. Secondary endpoints were: the prevalence of RLN injury, the frequency of total thyroidectomies, and the course of the RLN.

The study was approved by the Bioethics Committee of Wroclaw Medical University (Wroclaw, Poland).

All the patients included in the study were euthyroid; thyrotropin (TSH) and free thyroxine (fT4) levels were determined in each patient before surgery. Abnormal thyroid hormone levels and vocal cord paralysis were the exclusion criteria for the study. Both study groups (reoperations with and without IONM) included patients undergoing reoperations for recurrent goiter (multinodular goiter, Graves’ disease) many years after primary non-total thyroidectomies, reoperations due to compressive symptoms, suspicion of cancer, locoregional tumor control, or recurrent hyperthyroidism, as well as patients undergoing revisions of inadequate initial operations where thyroid carcinoma was recognized postoperatively.

A standard cervicotomy with excision of the existing scar was performed in each reoperative thyroid procedure. Different approaches to the remnant thyroid tissue were used. Most often we used a lateral approach, between the strap muscles and the sternocleidomastoid muscle, to reach the remnant thyroid tissue. This technique allowed the surgeon to reach the thyroid gland and expose the RLN through a previously undissected plane. A low anterior approach was used in reoperations with a huge retrosternal goiter.

In the non-monitored thyroid reoperations the RLN was only visually identified; the inferior thyroid artery, Zuckerkandl’s tubercle or the laryngeal entry point were the landmarks in visual RLN identification. In most cases we used palpation as an adjunct to visual identification of the RLN, but it was limited in same cases by the scar from the initial operation. In every case we started the approach to the RLN by mobilizing the inferior pole of the thyroid enough to roll the thyroid lobe medially.

In all the monitored thyroid reoperations, intermittent neuromonitoring with a NIM 3.0 nerve monitoring system (Medtronic, Minneapolis, MN, USA) was employed. A handheld bipolar stimulating probe was used with a current amplitude of 1–2 mA and 3 Hz impulses of 200 ms each for 1–2 s. Usually we mapped the RLN with 2 mA; 1 mA or less was used to confirm the course of the nerve or to distinguish the nerve from other tissues. The electromyographic (EMG) signal was obtained using surface electrodes integrated into the endotracheal tubes (NIM Flex EMG tubes, Medtronic); in female patients 7 mm endotracheal tubes were used, and in males 7.5 mm tubes were used. Neuromonitoring was carried out in accordance with the recommendations of the International Neuromonitoring Study Group [12]. The reoperations started with vagal stimulation (V1) on the operated side of the thyroid. Before removing the thyroid lobe, the RLN was usually identified by mapping techniques 2 mA (R1). Then, after the removal of the thyroid lobe, RLN conduction (R2) and the whole path of reflex including vagal nerve conduction was confirmed (V2). Laryngological examinations were performed in all patients preoperatively (L1) and two days after the thyroidectomy (L2). Where recurrent laryngeal nerve palsy was found, these examinations were repeated after 3, 6, and 12 months, or until the full recovery of the vocal cords.

Both the identification of the RLN and the number of nerve injuries were calculated in relation to the number of RLNs at risk of injury. The types of thyroid resection were categorized into one of four groups: thyroidectomies, near-total thyroidectomies, Dunhill operations and subtotal thyroid operations (more than 1 cm^3^ of remnant tissue on both sides).

All the thyroid procedures were performed by a team of three surgeons (average age 41), who annually perform 50–100 thyroid operations. These surgeons started to use IONM for the first time in 2012 diring both primary and secondary thyroid operations.

Photos or videos were taken to record the course of the RLN in each of the reoperative thyroid procedures.

## Results

From January 2011 to December 2014, 632 patients underwent thyroid surgery at the Wroclaw Medical University Department of General, Gastroenterological and Endocrine Surgery; (236 of them with IONM and 396 of them with visualization alone). Among them were 61 thyroid reoperations (105 RLNs at risk); 37 of these procedures (60 RLNs at risk) used IONM and 24 of them (45 RLNs) used visualization alone. The baseline demographic and intraoperative characteristics of patients in this study is shown in Table 1.

**Table 1:** Thyroid reoperations: the patients’ demographic and intraoperative characteristics

	Thyroid reoperations		
	Non-monitored group (visual RLN identification)	Monitored group (RLN identification with IONM)	*p value*
Number of patients	24	37	–
RLNs at risk	45	60	–
Males	3 (12.5%)	5 (13.5%)	0.909**
Females	21 (87.5%)	32 (86.5%)	
Age (years)	57.83 ± 14.43	55.75 ± 12.35	0.9252*
Volume of thyroid gland (ml)	29 ± 12.7	34 ± 8.9	0.4657***
Recurrence of goiter	18 (75%)	31 (84%)	0.399**
Multinodular goiter/thyroiditis	14	24	
Toxic goiter	4	–	
Papillary thyroid cancer	–	5	
Reoperation due to thyroid carcinoma	6 (25%)	6 (24%)	
Papillary carcinoma	6	4	
Follicular carcinoma	–	2	
The extent of primary thyroid operation:			0.1015**
Bilateral:	21 (87.5%)	30 (81%)	
Subtotal thyroidectomy	18 (75%)	20 (54%)	
Near total thyroidectomy	3 (12.5%)	3 (8%)	
Dunhill operation:	–	7(19%)	
Unilateral:	3 (12.5%)	7(19%)	
Lobectomy	3 (12.5%)	7(19%)	
Retrosternal goiter	6 (25%)	10 (27%)	0.5259**
Displacement, narrowing of trachea	10 (42%)	26 (70%)	0.0854**

*RLN* recurrent laryngeal nerve, *IONM* intraoperative nerve monitoring

*t-test, ** Fisher’s exact test, *** Mann–Whitney *U*-test

### Primary endpoint analysis

The rate of RLN identification was calculated in relation to the number of nerves at risk during the procedures. In the non-monitored group (24 operations, 45 RLNs at risk) only 20 RLNs (44.4%) were identified. In the IONM group (37 operations, 60 RLNs at risk) 55 RLNs were identified (91.6%). This difference is statistically significant (*p* < 0.001).

**Fig. 1:**
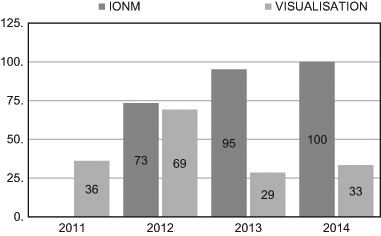
The rate of RLN identification (%) in reoperations with IONM vs. visualization alone in 2011–2014 year

In 2011, before the use of IONM was introduced at the authors’ department, the rate of RLN identification by visualization alone was 36% in reoperative procedures. In 2012, the first year of the authors’ use of IONM, the rate of RLN identification in reoperations with IONM was 73.4%, and in 2013 and 2014 it increased to 95.2 and 100%, respectively. The rate was greater each year, but the rising trend was not significant (Pearson correlation: *r* = 0.938, *p* = 0.799). In non-monitored reoperative thyroid surgery the rates of RLN identification from 2012 through 2014 were 69.2, 28.6, and 33.3%, respectively. This indicates that the use of IONM did not significantly influence surgical skills in operations in which IONM wasn’t used (*p* = 0.19) (Table 2, Fig. 1).

**Table 2:** Thyroid reoperations; the rate of RLN identifications: Visualization vs. IONM

	RLN visual identification			RLN identification with IONM			
Year	Number of patients	RLNs at risk (100%)	RLNs identification	Number of patients	RLNs at risk (100%)	RLNs identification	*p v*alue Chi- squared test
2011	11	22	8(36%)	–	–	–	
2012	7	13	9(69.2%)	9	15	11(73.3%)	*p = 0.8106*
2013	4	7	2(28.6%)	13	21	20(95.2%)	*p = 0.0002*
2014	2	3	1(33.3%)	15	24	24(100%)	*p < 0.0001*
Total	24(100%)	45(100%)	20(44.4%)	37(100%)	60(100%)	55(91.6%)	*p < 0.001*

In 2012 the rate of RLN identification was not significantly different between traditional and IONM group (69.2 vs. 73.3%, *p* = 0.8106). In 2013 and 2014 the rate of RLN identification was significantly higher in IONM group (2013: 28.6 vs. 95.2%, *p* = 0.0002; 2014: 33.3 vs. 100.0%, *p* < 0.0001) according to chi-squared test (Table 2).

**Fig. 2:**
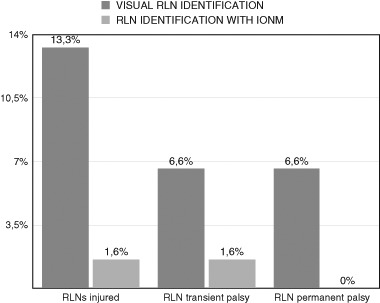
Postoperative RLNs injured with and without IONM

**Fig. 3:**
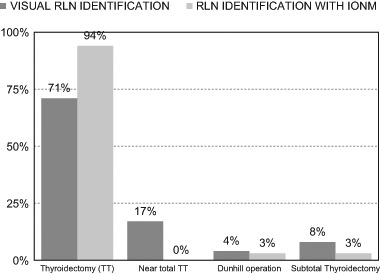
Type of thyroid surgery with and without IONM

In the reoperations using IONM, 1 mA stimulation was sufficient to map the RLN in 27% of the procedures, while 2 mA stimulation, which is used in heavily scarred operating fields where the RLN is imbedded or adherent to other tissues after previous operations, was needed in 60%. In 13% of the cases was the course of the nerve visualized before it was confirmed by IONM, making mapping unnecessary. (Figs. 2 and 3)

### Secondary endpoints analysis

#### The rate of RLN injury

In the non-monitored group six nerves (13.3%) were injured; three of the injuries (6.6%) were transient and three (6.6%) were permanent. The injury was unilateral in four patients. The one bilateral injury (in 2011, one year before IONM was introduced) was transient. After two months one of the two injured nerves regained function. In the IONM group only one unilateral transient injury (1.6%) was observed, in the second year of IONM use (Table 3)

**Table 3:** Postoperative complications with and without IONM

	Non-monitored group (visual RLN identification)	Monitored group (RLN identification with IONM)	*p* value
Number of patients	24 (100%)	37 (100%)	–
RLNs at risk	45 (100%)	60 (100%)	–
RLNs injured	6 (13.3%)	1 (1.6%)	–
Transient	3 (6.6%)	1 (1.6%)	p = 0.185**
Permanent	3 (6.6%)	–	*p = * ***0.042*** **

The bold values are statistically significant

*RLN* recurrent laryngeal nerve, *IONM* intraoperative nerve monitoring

** Fisher’s exact test

There were no statistically significant differences between the two groups (with and without IONM) in terms of either transient (*p* = 0.185) or permanent paresis (*p* = 0.042).

#### The course of the nerve

A non-anatomical course of the nerve was observed in 80% of the reoperative procedures. It was found that 57% of the nerves were imbedded in extensive scarring and fibrosis left by the initial operations. These nerves were usually mapped during the reoperations. RLNs adherent to the lateral capsule of the goiter were observed in 15%; in 8% of the procedures the nerves were fixed to the inferior part of the goiter, in particular in cases of retrosternal goiter. A typical course of the nerve was observed in only 20% of the reoperations, usually many years after the first intervention.

#### The type of thyroid operation

In the IONM group, 94% of the patients underwent total resection; there were no near-total operations. One patient (3%) underwent subtotal resection, and one (3%) had one lobe totally removed and the second partially removed (a Dunhill operation). In the group reoperated without IONM, partial thyroidectomies were performed in six patients (29%) and total thyroidectomies in 17 (71%) (Table 4). The differences in the range of surgery were statistically significant (*p* = 0.043)

**Table 4:** Type of thyroid surgery with and without IONM

	Non-monitored group (visual RLN identification)	Monitored group (RLN identification with IONM)	*P value*
Number of reoperations	24 (100%)	37 (100%)	–
Total resections	17 (71%)	35 (94%)	*p = * ***0.043*** **
Near-total resections	4 (17%)	–	
Dunhill operations	1 (4%)	1 (3%)	
Subtotal thyroidectomy	2 (8%)	1 (3%)	

The bold values are statistically significant

*RLN* recurrent laryngeal nerve, *IONM* intraoperative nerve monitoring

** Fisher’s exact test

#### The duration of reoperations

The mean duration of the reoperations with IONM was 113 min. This was longer than the mean time of the reoperations without IONM (108 min), but the difference was not statistically significant (*p* = 0.818)

## Discussion

Injury of the RLN during thyroidectomy is always a major complication leading to the vocal cord pulsy that can significantly influence the quality of life. In recent years, because of an easy access to US-guided FNAC, many more thyroidectomies are performed. As a consequence, RLN pulsy has become the main cause of medical ligitations in many countries in which Gambardella et al. pointed out in his recent review about unintentional recurrent laryngeal nerve injuries following thyroidectomy [15]. The authors offered medical practice recommendations aimed at reducting the risk of malpractice lawsuits and judgments against surgeons. They emphasized that visualization, dissection, and intraoperative nerve monitoring have reduced the incidence of unintentional RLN injury to 1–2% in tertiary referred centers. According to Gambardella et al., this complication is predictable but not preventable, and should not be considered as a “surgeon mistake”, but rather an unavoidable complication [15]. This problem of unintentional recurrent laryngeal nerve injuries is particularly significant in thyroid reoperations.

Reoperative thyroid surgery is associated with a higher incidence of complications–both RLN palsy and hypoparathyroidism. These operations are difficult for inexperienced surgeons and residents, especially because of scarring and fibrosis from the initial operation. Even skilled surgeons with a good knowledge of RLN anatomy and good knowledge of various techniques of approaching the remnant thyroid tissue often have problems identifying the RLN and preventing injury to it. The average reported rates of RLN injury in reoperative procedures–12.5% for transient palsy and 3.8% for permanent injury–are significantly higher than in primary thyroid operations [16–21].

Out of the secondary operations the most difficult seems to be this after primary partial resection of the thyroid gland. As Conzo et al. pointed out, the rate of complications after completion of thyroidectomy, after loboisthmusectomy due to neoplasia folliculare with carcinoma in histopatological examination, were 3.1%. Transient and permanent RLN palsy were observed only in 0.3% and 0.3% RLNs subsequently [22]. Therefore the importance of having the first radical treatment on the thyroid gland seems to be of key to avoid complications during reoperation. However, we still have patients that need difficult re-do surgery and surgeons should use all the resources to prevent RLN from inadvertent injury.

Since 1966, when Shedd introduced neuromonitoring of the recurrent laryngeal nerve in thyroid surgery [23], there has been interest in the question of whether this technique could minimize the risk of RLN injury during thyroidectomy. It took many years to address this question, because initially IONM was not standardized, and the early data were of limited value [24, 25]. Nowadays IONM of the RLN and the external branch of the superior laryngeal nerve (EBSLN) is standardized [12, 13], which means the results of IONM use can be compared and analyzed [26, 27]. But when considering the results of IONM use in thyroid surgery, particularly in thyroid reoperations, we have to emphasize that there are some limitations in most published reports about IONM. The main limitations of the present study on thyroid reoperations are the small number of patients involved, the lack of homogeneity among the patients undergoing thyroid reoperations, their indications for the procedures and the extent of surgery (ranging from a lobectomy to a total resection). Nevertheless, we have tried to assess the use of IONM in reoperative thyroid surgery.

Identification of the RLN is basic to all thyroid operations. In 1994 Jatzko showed that recurrent nerve paralysis is a less frequent complication when the nerve is identified [10]. In primary thyroid operations, good knowledge of the anatomical variants of the RLN and years of experience with thyroid procedures allow surgeons to identify the nerve in most cases. But it is much more difficult to identify the RLN in reoperative thyroid surgery. There are studies suggesting that the use of IONM improves nerve identification rates. In initial thyroid operations, the rate of RLN identification without IONM is about 90%, as opposed to 99.3% with IONM [28–31]. The high rate of RLN identification when using IONM is possible because of mapping, which allows surgeons to locate the nerve before visual confirmation. The nerve is mapped out in the paratracheal region through probe stimulation, and then visually identified through dissection directed by this mapping [32].

In the authors’ experience the rate of RLN identification in reoperative thyroid surgery without the use of IONM was very low–44.44%–but with IONM the rate of RLN identification was 91.66%, and this difference is statistically significant. Interestingly, from year to year we observed steady increases in the rate of RLN identification with IONM, from 73.4% in 2012 to 100% in 2014. The same trend was not observed in operations using visual RLN identification alone. The high rate of RLN localization was possible thanks to mapping with 2 mA in 60% of the nerves, which means that in more than half of the reoperations the nerve was first localized by mapping in scarred fields, and then identified visually. A study by Barczynski et al. on reoperative thyroid surgery using IONM showed that 20% of the nerves were identified with neuromonitoring before visual exposition; moreover, with monitoring it was possible to find twice as many ramified nerves than in reoperations without the use of IONM (*p* = 0.001) [33]. The low rate of nerve identification in reoperations without monitoring confirms that even skilled surgeons may be unable to find the RLN in an operating field altered by previous surgery. Using IONM, we observed a non-anatomical RLN course in 80% of the cases; in 57%, the nerves were embedded in extensive scarring and fibrosis after the first operation. Another problem observed in reoperations was that the RLN adhered to the lateral capsule of the goiter in 15% of the cases; 8% of the nerves were fixed to the inferior part of the goiter. A typical course of the nerve was observed only in 20% of the cases. IONM allowed these variations and pitfalls to be easily recognized, and the distorted nerves could be safely dissected and protected [34]. Moreover, in scarred fields IONM is helpful in distinguishing nerves from other structures, such as blood vessels or fibrosis [35].

If the RLN is identified almost in 100% in reoperative thyroid surgery, the rate of complications should be lower. But this hypothesis is very difficult to confirm. Firstly, reoperative thyroid surgery comprises only 10% of all thyroid operations, since total thyroidectomy is the preferred procedure at most institutions. Secondly, the incidence of permanent and transient RLN injury is relatively low, and in order to achieve adequate statistical power, studies with a sufficiently large sample size are needed [36]. Moreover, there are many factors that could influence the rate of RLN injury, such as the surgeon’s expertize, the histological nature of the reoperative disease, the type and extent of thyroid resection. Additionally, not all studies conduct preoperative and postoperative laryngoscopy as a mandatory tool for vocal mobility assessment [36–38]. Most of these limitations affect the present study as well. The rates of transient (1.6%) and permanent (0%) injury were lower in the IONM group in comparison with the non-monitored group (6.6% for both types) but the differences were not statistically significant (transient injury: *p* = 0.185; permanent injury: *p* = 0.042).

In the available literature there are only a few publications concerning the prevalence of RLN injury while using IONM in thyroid reoperations [33, 39–41]. Barczyński et al. compared reoperations with and without neuromonitoring in a retrospective study in 2014, and found that neuromonitoring reduced the prevalence of RLN injury in thyroid reoperations to a statistically significant degree compared with reoperations in which the nerve was only visually identified (*p* = 0.001). With IONM, transient injuries were found in 13 nerves (2.6%) and permanent injuries in seven (1.4%), as opposed to 52 (6.3%) and 20 (2.4%), respectively, without IONM [33].

Chuang and Huang recommended the routine use of IONM in thyroid and parathyroid reoperations because the rate of RLN injury with IONM was 1.43%, as opposed to 20% without monitoring (*p* = 0.0164) [39]. On the other hand, Yarbrough et al. found that intraoperative monitoring of the RLN can be performed safely during reoperative neck surgery, but did not decrease RLN complications; in their study the rate of RLN complications was similar in both groups (1.9% with IONM vs. 1.7% without IONM) [40]. Similarly, Alesina et al. did not observe significant differences in the prevalence of RLN injuries in operations with and without the use of IONM (*p* = 0.1) [41]. The studies by Yarbrough et al. [40] and Alesina et al. [41] were both based on relatively small groups of patients, and statistical validation of small differences in the prevalence of RLN injury requires more RLNs at risk.

The last point of the present study was the type/extent of the reoperative thyroid procedures performed. A second operation should always aim for completeness in order to spare the patient any further surgery, each of which would risk RLN injury. Neuromonitoring allowed us to remove all the remnant tissue in 94% of the patients, with no near-total resections. This was possible because IONM permits the surgeon to remove remnant tissue close to the laryngeal entry point, where the nerve is at a high risk of injury [40]. Only 71% of the operations performed without IONM were total resections. Without adequate nerve identification, partial resections were performed in 29% of the cases. The difference in terms of the extent of surgery were statistically significant (*p* = 0.043).

It is worth pointing out that the use of IONM can change the surgical strategy during reoperations. During a thyroid operation, when a loss of signal is observed on the side of the neck operated on first, a staged thyroidectomy should be considered in order to prevent bilateral nerve injuries. Staged thyroidectomies have been gaining acceptance among surgeons since IONM was introduced, and it is particularly important in reoperations [42]. The present authors did not perform any staged thyroidectomies in the IONM group, because no loss of signal was observed on the side operated on first.

There is currently no hard evidence that IONM can diminish the prevalence of permanent vocal fold palsy, but more than 90% of the respondents in the most recent international survey on the identification and neural monitoring of the EBSLN during thyroidectomy emphasize their confidence in IONM, and listed reoperative thyroid cases as the top indication–far higher than any other clinical situation–for the use of this technique during thyroid surgery [43].

In the end we have to note that there is no consensus regarding the utylity of IONM and its role is still being evoluated. It is not a standard of care in majority of cantries. However, our data and experience confirmed that its application should be considered for high-risk patients, especially in thyroid reoperation. Without a doubt, IONM protects from bilateral palsy; in case of loss of signal on the first operated side the other side should not be operated [12, 15].

## Conclusions

Thyroid reoperations performed with IONM entailed a significantly improved RLN identification rate and were significantly more radical than reoperations conducted with visualization alone.
